# Association of soluble T cell immunoglobulin domain and mucin-3 (sTIM-3) and mac-2 binding protein glycosylation isomer (M2BPGi) in patients with autoimmune hepatitis

**DOI:** 10.1371/journal.pone.0238540

**Published:** 2020-12-21

**Authors:** Kiyoshi Migita, Minoru Nakamura, Yoshihiro Aiba, Hideko Kozuru, Seigo Abiru, Atsumasa Komori, Yuya Fujita, Junpei Temmoku, Tomoyuki Asano, Shuzo Sato, Makiko Furuya, Atsushi Naganuma, Kaname Yoshizawa, Masaaki Shimada, Keisuke Ario, Tomohiko Mannami, Hiroshi Kohno, Toshihiko Kaneyoshi, Takuya Komura, Hiromasa Ohira, Hiroshi Yatsuhashi

**Affiliations:** 1 Clinical Research Center, Nagasaki Medical Center, Nagasaki, Japan; 2 Department of Rheumatology, Fukushima Medical University, Fukushima, Japan; 3 National Hospital Organization, Takasaki Medical Center, Takasaki, Japan; 4 National Hospital Organization, Shinsyu-Ueda Medical Center, Ueda, Nagano, Japan; 5 National Hospital Organization, Nagoya Medical Center, Nagoya, Aichi, Japan; 6 National Hospital Organization, Ureshino Medical Center, Ureshino, Saga, Japan; 7 National Hospital Organization, Okayama Medical Center, Okayama, Okayama, Japan; 8 National Hospital Organization, Kure Medical Center, Kure, Hiroshima, Japan; 9 National Hospital Organization, Fukuyama Medical Center, Kanazawa, Ishikawa, Japan; 10 National Hospital Organization, Kanazawa Medical Center, Kanazawa, Ishikawa, Japan; 11 Department of Gastroenterology, Fukushima Medical University, Fukushima, Japan; University of Tsukuba, JAPAN

## Abstract

**Background:**

Autoimmune hepatitis (AIH) is a disorder of unknown etiology in which immune-mediated liver injury progress to cirrhosis or hepatocellular carcinoma (HCC). The aim of the present study was to determine whether circulating soluble TIM3 (sTIM3) is elevated in patients with AIH patients and whether sTIM-3 levels are associated with clinical parameters of AIH.

**Methods:**

We enrolled 123 Japanese patients with AIH who were identified from the National Hospital Organization–AIH-liver–network database, as well as 32 patients with chronic hepatitis C (CHC), 30 patients with primary biliary cholangitis (PBC) and healthy control subjects. Serum sTIM-3 concentrations were quantified by ELISA.

**Results:**

Serum levels of sTIM-3 were significantly higher in AIH patients (median 4865 pg/ml; [interquartile range (IQR); 3122–7471]) compared to those in CHC (1026 pg/ml [IQR: 806–1283] *p*<0.001), PBC (2395 pg/ml [IQR: 2012–3422] *p*<0.001) or healthy controls (1285 pg/ml [IQR: 1098–1812] *p*<0.001). In AIH group, serum sTIM-3 were correlated with alanine aminotransferase (ALT), or total bilirubin (TB) and negatively correlated with serum levels of albumin (Alb). Serum levels of sTIM-3 were also strongly correlated with Mac-2 binding protein glycosylation isomer (M2BPGi) levels, but did not correlate with the histological grade of liver fibrosis. Steroid treatment of AIH patients significantly reduced serum sTIM-3 levels (2147±623pg/ml versus 1321±378pg/ml, *p*<0.001).

**Conclusions:**

Circulating sTIM-3 levels were elevated in AIH patients and are associated with AIH disease activity and AIH-related liver damage. These findings indicate that serum sTIM-3 correlated with disease status of AIH and could be useful biomarkers to detect autoimmune-mediated liver injury. Our data suggest a possible link between the TIM-3/GAL-9 pathway and AIH severity or phenotype, and further investigations of the TIM-3 pathway and AIH pathophysiology is warranted.

## Background

T cell immunoglobulin domain and mucin domain containing molecule 3 (TIM-3) is expressed on the surface of terminally differentiated T cells and negatively regulates the T cell response by inducing T cell apoptosis after binding its ligand, galectin-9 [1]. Recent studies have shown that TIM-3 also plays an important role in the regulation of macrophages, monocyte, and dendritic cells [2]. Dysregulation of TIM-3 expression on immune cells may lead to excessive or inhibited pro-inflammatory or autoimmune responses under various pathogenic conditions [1]. Autoimmune hepatitis (AIH) is a chronic inflammatory liver disease with unknown pathogenesis, characterized by the elevation of transaminases and IgG, the presence of autoantibodies and lymphoplasmacellular infiltrates in liver [3]. The TIM-3/galectin-9 interaction is presumed to be involved in immune-mediated liver diseases including AIH [4]. Blocking the TIM-3/galectin-9 pathway leads to the exacerbation of acute hepatitis in mice through cytokine (IFN-γ) generation in experimental models [5]. It was presumed that the dysregulated TIM-3 pathway may be the impaired in patients with AIH [6]. A better understanding of the pathogenic role of TIM-3 pathway may lead to new insights into the pathogenesis of autoimmune diseases including AIH.

Tim-3 can be shed from the cell surface by a disintegrin and metalloproteinase with a thrombospondin type 1 motif, member 13 (ADAMTS13) or ADAMTS17-mediated cleavage of the TIM-3 stalk region, resulting in a soluble from that is elevated in the sera of patients with autoimmune diseases [7, 8]. We hypothesized that the Tim-3 axis is impaired in AIH. We investigated the serum levels of sTIM-3 in Japanese patients with AIH and analyzed the associations between sTim-3 and clinical parameters of AIH.

## Materials and methods

### Study population

Patients with well-documented and untreated AIH were enrolled from the National Hospital Organization (NHO)-AIH-liver-network database, a multicenter registry for Japanese patients with AIH [9]. The diagnosis of AIH was made according to the diagnostic criteria defined by International Autoimmune Hepatitis Group [10]. Patients were excluded from the study if there was histological evidence of cholangitis or non-alcoholic steatohepatitis. Patients positive for hepatitis B virus surface antigen or HCV RNA were also excluded. Patients with other causes of liver disease, such as excess alcohol or drug use, were excluded based on reviews of their appropriate history and investigations. As controls, patients with chronic hepatitis C (untreated CHC, n = 32; female/male = 16/16; mean) were enrolled. The baseline characteristics of CHC patients are listed as median age 57.5 years (median) 51.1–62.1 (IOQ); aspartate aminotransferase; 34.0 IU/L (median) 28.3–49.8 (IOQ), alanine transaminase; 40.0 IU/L (median) 25.0–74.0 (IOQ, total bilirubin; 0.7 mg/dL (median) 05–0.9 (IOQ). As controls, 27 healthy subjects (11 males, 16 females, median age 40.3 years IOQ, 35.6–42.4) and as controls for autoimmune disease, PBC patients (n = 30; female/male = 25/5) were included. The baseline characteristics of PBC patients are listed as median age was 57.0 years 38.0–67.0 (IOQ), alkaline phosphatase (ALP); 592 IU/L (median) 255–1223 (IOQ); immunoglobulin M (IgM); 249 mg/dL (median) 44–736 (IOQ). The study was approved by the Ethics Committee of the NHO Central Internal Review Board and participating NHO liver-network hospitals (H30-0313001). Written informed consent was obtained from each individual. All research was performed in accordance with relevant guidelines/regulations.

### Histological assessments

Liver biopsy and laboratory tests were obtained at baseline prior to treatment. In the histological diagnosis of AIH, each specimen was assessed for inflammatory grading including the degree of portal inflammation, presence of interface hepatitis, and the degree of parenchymal inflammation, as well as the stage of fibrosis (0, absent; 1, expansion of fibrosis to parenchyma; 2, portal–central or portal–portal bridging fibrosis; 3, presence of numerous fibrous septa; and 4, multi-nodular cirrhosis) according to the criteria of Desmet et al [11]. Cirrhosis was diagnosed histologically when a loss of normal lobular architecture, reconstruction of hepatic nodules, and the presence of regenerative nodules were observed.

### Enzyme-linked immunosorbent assay for sTIM-3

Serum concentrations of soluble TIM-3 were measured using human enzyme-linked immunosorbent assay kit (R&D Systems, Minneapolis, MN, USA) according to the manufacturer’s instruction.

### Measurement of mac-2 binding protein glycosylation isomer (M2BPGi)

Serum M2BPGi level was directly measured with the HISCL^™^ M2BPGi^™^ reagent kit (Sysmex, Kobe, Japan) using an automatic immunoanalyzer HISCL‐5000 (Sysmex, Hyogo, Japan). M2BPGi levels were indexed using the following equation: Cut‐off Index (C.O.I.) = ([M2BPGi]sample‐[M2BPGi]NC)/([M2BPGi]PC)‐[M2BPGi]NC), where [M2BPGi]sample represents the M2BPGi count of the serum sample, PC is positive control, and NC is negative control [12].

### Statistical analysis

Correlations between continuous variables were analyzed by the Spearman’s correlation test. Results for non-normally distributed continuous variables were summarized as mean or medians (interquartile ranges IQR) and were compared by the Mann-Whitney U test. Paired data were analyzed by non-parametric tests using the Wilcoxon signed-rank test for the comparison of paired data.

## Results

### Serum levels of sTim-3 in AIH patients and healthy subjects

**Fig 1:**
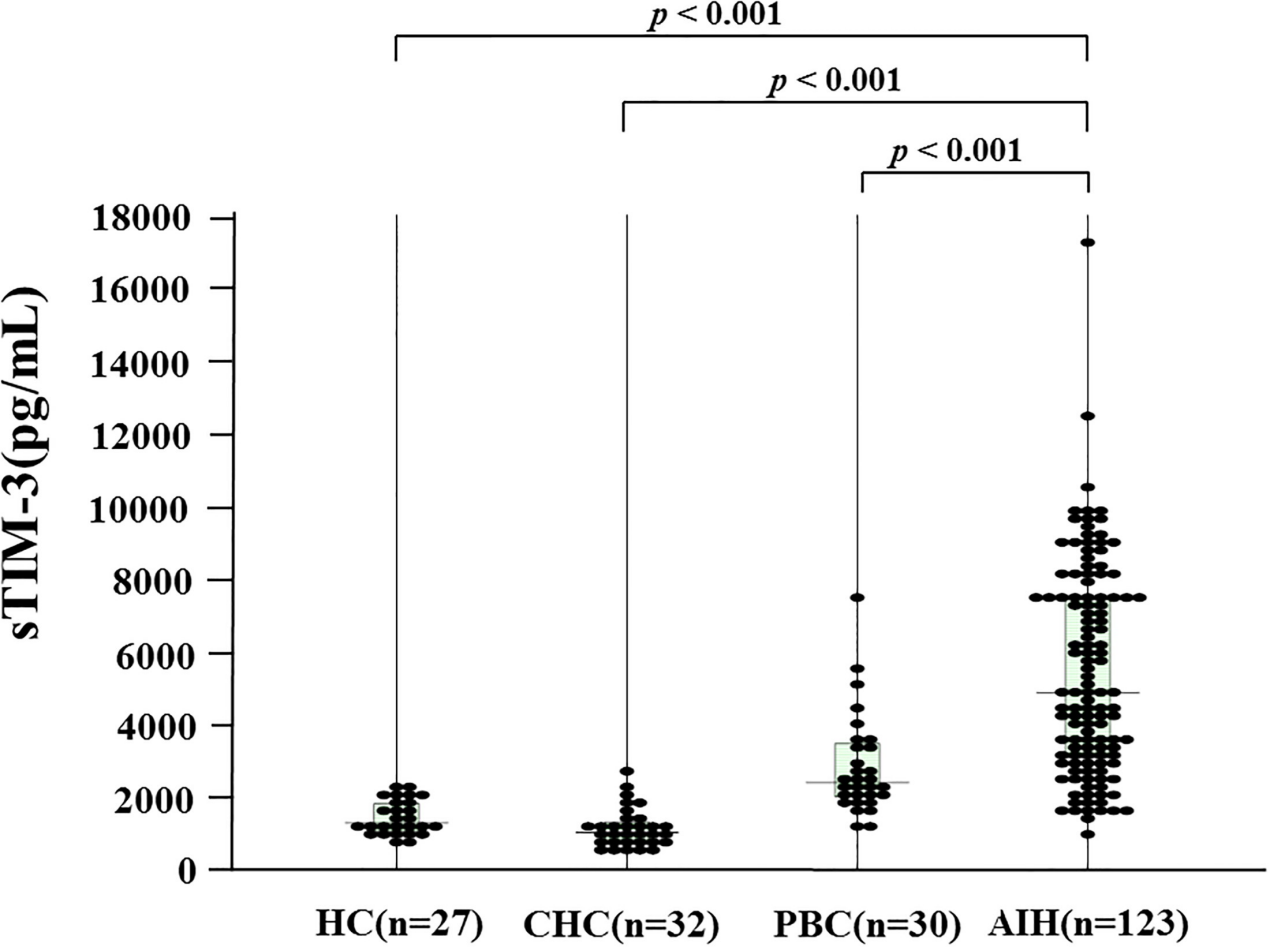
Serum levels of sTIM-3 in AIH patients (n = 123), patients with chronic hepatitis C (HCV, n = 32), primary biliary cholangitis (PBC, n = 30) and healthy subjects (n = 27). The vertical lines indicate the range and the horizontal boundaries of the boxes represent the first and third quartiles. Results were compared by non-parametric Mann–Whitney *U*-test.

Table 1 shows the demographic data of the 123 AIH patients included in this study. Among the 123 patients with type-1 AIH, 81 (70.4%) were positive (>1:40) for anti-nuclear antibodies. Ten patients (8.1%) had liver cirrhosis at the time of diagnosis. We compared serum sTim-3 levels in AIH patients (n = 123), patients with chronic hepatitis C (CHC, n = 32), primary biliary cholangitis (PBC, n = 30) and healthy subjects (n = 27). As shown in Fig 1, serum levels of sTIM-3 were significantly higher in AIH patients (median 4865 pg/ml; [interquartile range (IQR); 3122–7471]) compared with those in CHC patients (1026 pg/ml [IQR: 806–1283] *p*<0.001), PBC patients (2395 pg/ml [IQR: 2012–3422] *p*<0.001) or healthy patients (1285 pg/ml [IQR: 1098–1812] *p*<0.001).

**Table 1:** Baseline characteristics of 123 patients with AIH.

Characteristics	n = 123
Female, n/total(%)	102(82.9)
Age, y, mean ±SD	59.0(12.8)
median(IQR)	60.0(51.0–68.0)
Biochemistry	
AST, IU/L, median (IQR)	343 (180–669)
ALT, IU/L, median (IQR)	421 (223–853)
Total Bilirubin, mg/dl, median (IQR)	1.2 (0.8–4.3)
Albumin, g/dl, median (IQR)	3.9 (3.4–4.1)
IgG, mg/dl, median (IQR)	2053 (1634–2907)
Prothrombin time, %, median (IQR)	83.0 (72.6–93.9)
Serology	
ANA ≧ 1:40, n/total(%)	81 (70.4)
Cirrhosis, n/total(%)	10 (8.1)
IAIHG criteria	
Score, median (IQR)	16 (15.0–18.0)
Prednisolone use, n(%)	112 (91.1)
Immunosuppressant use, n(%)	17 (13.8)
Histology	
Fibrosis (N) F0/F1/F2/F3/F4	N = 100 3/46/20/27/4
Activity (N) A0/A1/A2/A3	N = 100 0/14/33/53

AST, aspartate aminotransferase. ALT, alanine aminotransferase.

IgG, immunoglobulin G. ANA, anti-nuclear antibody. IQR, interquartile range.

IAIHG, International Autoimmune Hepatitis Group.

### Relationships between serum sTIM-3 levels and liver function tests

**Fig 2:**
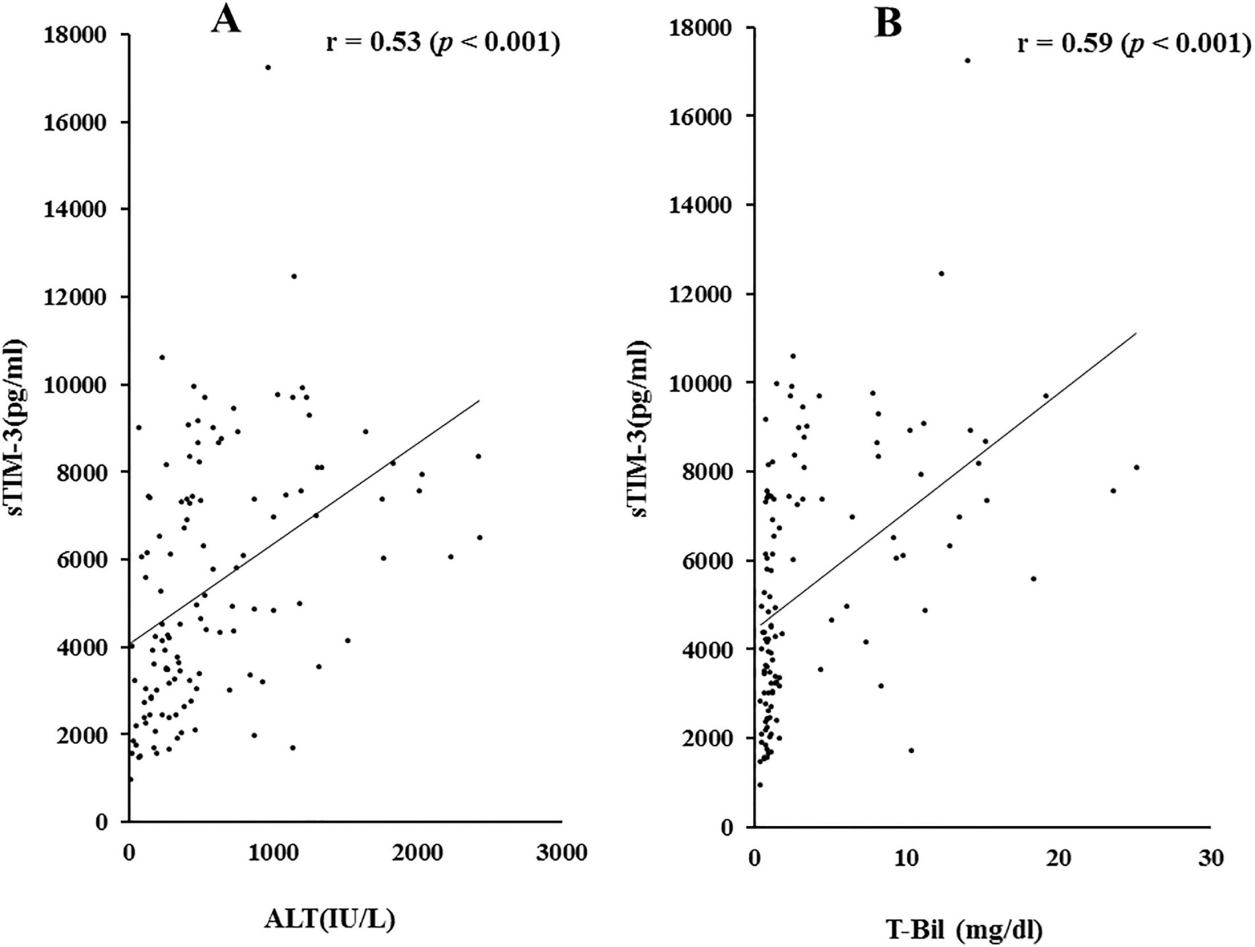
Correlations between serum sTIM-3 and ALT (A) or TB (B) levels in patients with AIH. Serum sTIM-3 significantly correlated with serum ALT and TB level. Statistics and regression line are represented by the solid line. ALT = alanine aminotransferase, TB = total bilirubin.

To investigate the relationship between sTIM-3 and clinical parameters, we examined the correlations between serum sTIM-3 and several liver function parameters. We detected significant correlations between sTIM-3 and alanine aminotransferase (ALT; r = 0.53 *p*<0.001) and total bilirubin (TB; 0.59 *p*<0.001) levels (Fig 2).

**Fig 3:**
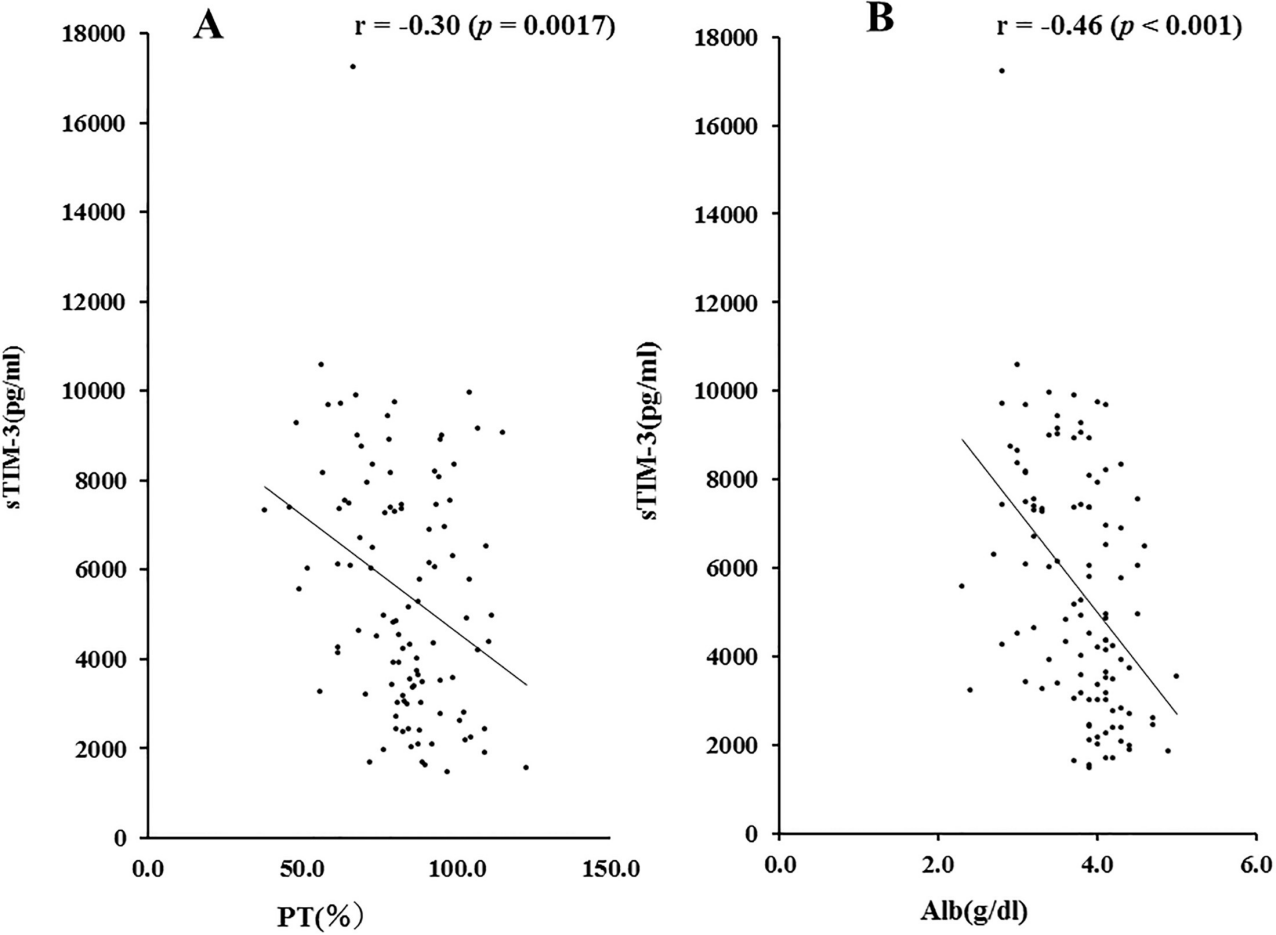
Correlations between serum sTIM-3 and PT time (A) or Alb (B) levels in patients with AIH. Serum sTIM-3 significantly negatively correlated with PT time and serum Alb level. Statistics and regression line are represented by the solid line. PT time = prothrombin time, Alb = albumin.

We also examined the correlations between sTIM-3 and prothrombin time (PT time %) and serum albumin levels, which are related to hepatic spare ability. As shown in Fig 3, there were significant negative correlations between circulating sTIM-3 and PT time (r = -0.30, *p* = 0.017) or serum Alb levels (r = -0.46, *p*<0.001).

**Fig 4:**
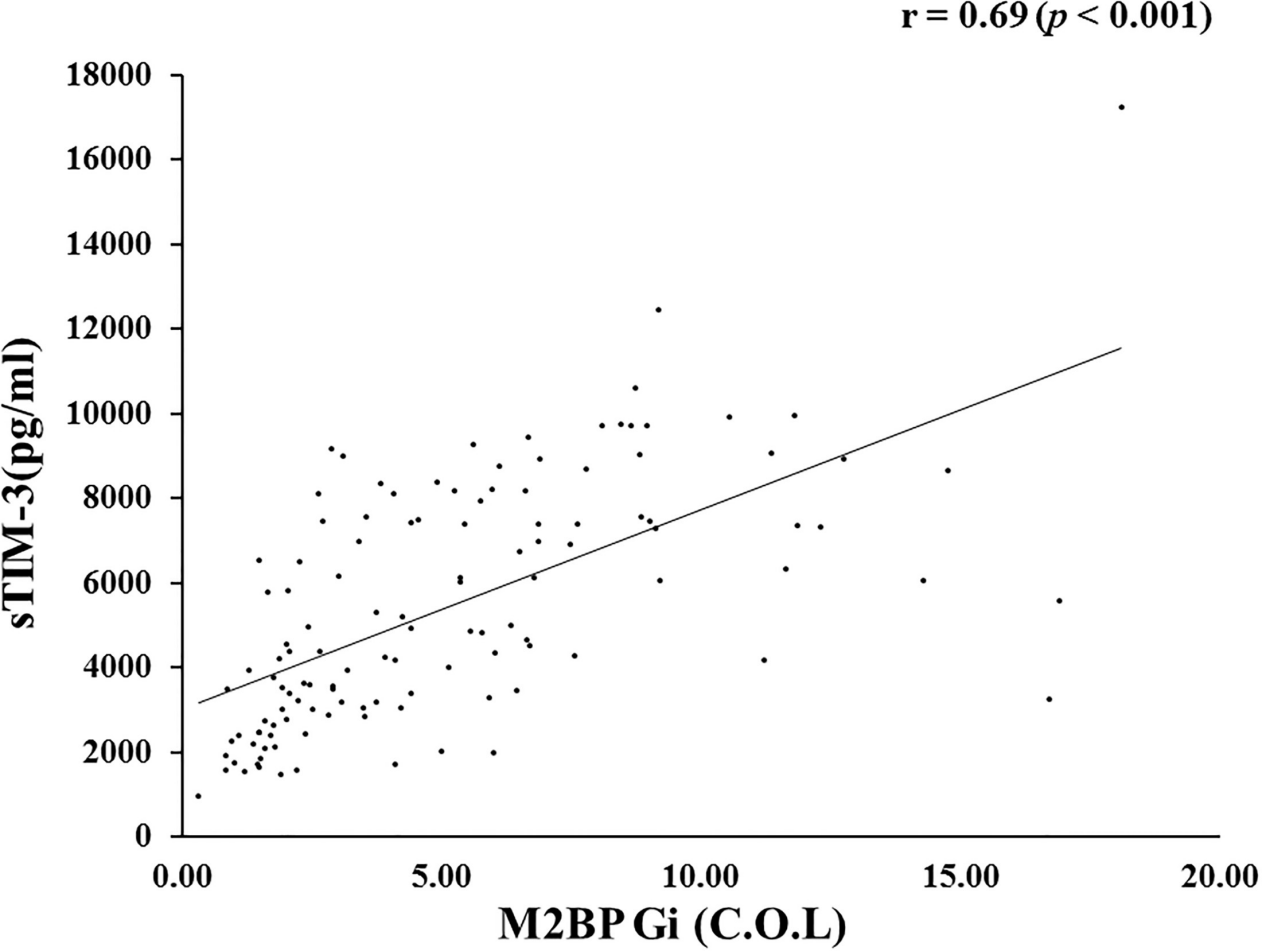
Correlations between serum sTIM-3 and M2BPGi in patients with AIH. Serum sTIM-3 significantly correlated with serum levels of M2BPGi. Statistics and regression line are represented by the solid line. M2BPGi = Mac-2 binding protein glycosylation isomer.

Interestingly, serum sTIM-3 levels were strongly correlated with Mac-2 binding protein glycosylation isomer (M2BPGi) levels (r = 0.69, *p*<0.001) in AIH patients (Fig 4).

**Fig 5:**
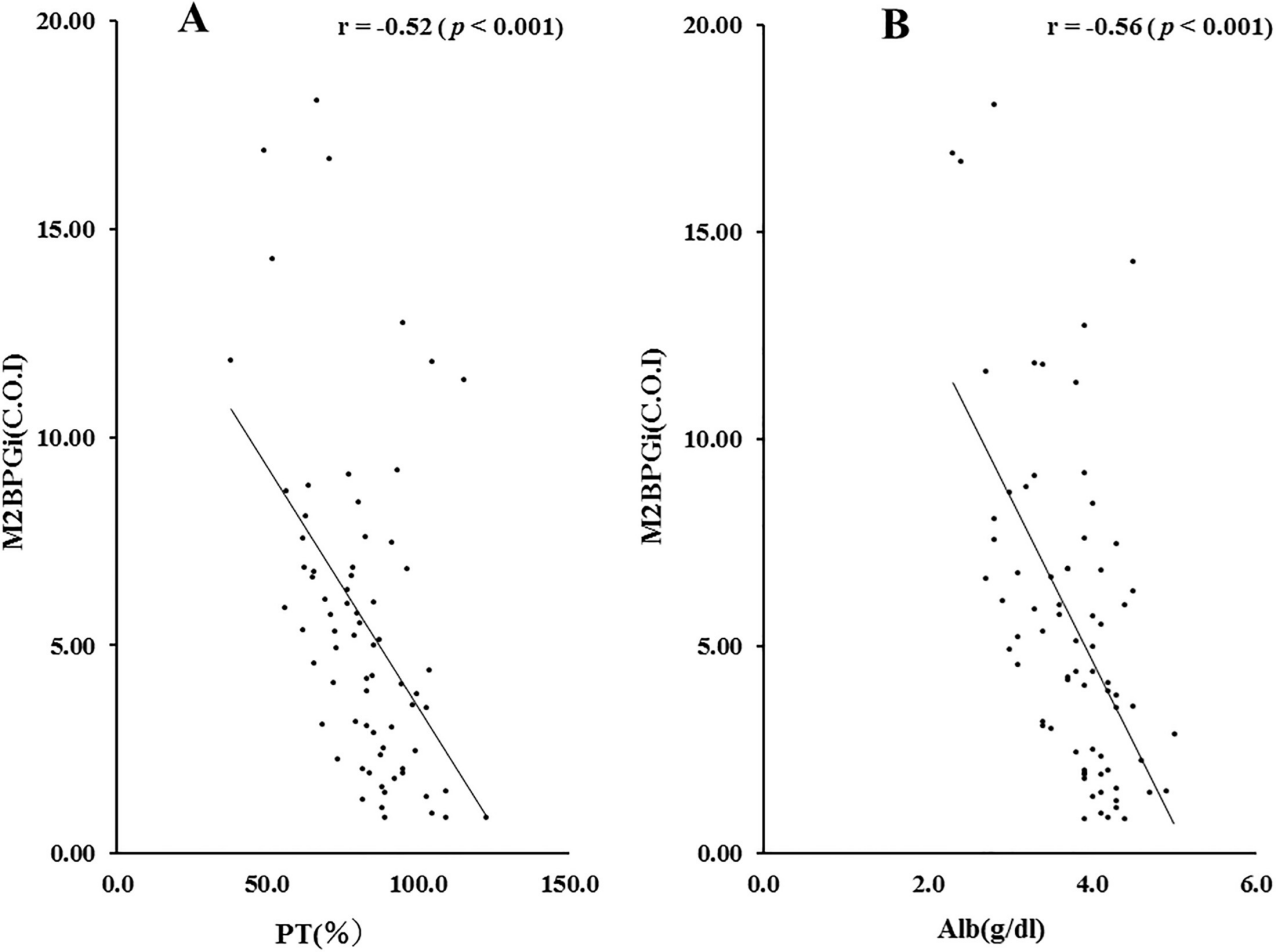
Correlations between serum levels of M2BPGi and PT time (A) or Alb (B) levels in patients with AIH. Serum levels of M2BPGi significantly negatively correlated with PT time and serum Alb level. Statistics and regression line are represented by the solid line. PT time = prothrombin time, Alb = albumin. M2BPGi = Mac-2 binding protein glycosylation isomer.

Therefore, we also investigated the correlations of M2BPGi levels and hepatic spare ability test. As shown in Fig 5, there were significant negative correlations between circulating M2BPGi and PT time or serum Alb levels in AIH patients.

**Fig 6:**
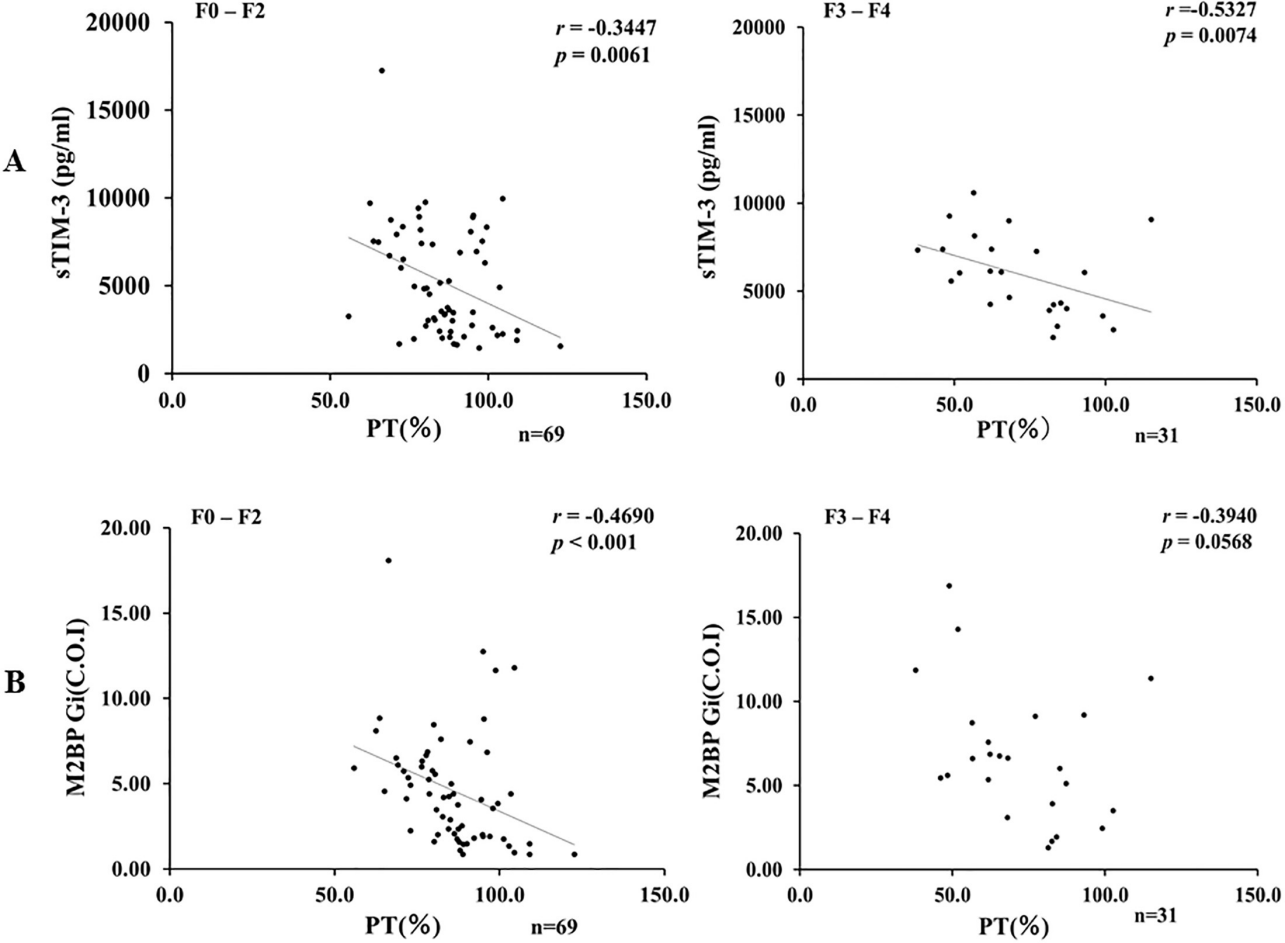
Correlations between serum levels of sTIM-3 (A) or M2BPGi (B) and PT time in AIH patients sub-grouped according to the stage of fibrosis (F0-F2 versus F3-F4). **A:** Serum levels of sTIM-3 were significantly negatively correlated with PT time regardless of the stage of fibrosis. **B:** There was no significant correlation between serum levels of M2BPGi and PT time in advanced liver fibrosis stage (F3-F4).

Although there was similar properties between sTIM-3 and M2BPGi concerning the correlations analysis with the liver function tests, there was one exception. In advance liver fibrosis stage (F3-F4), there was no significant correlation between circulating M2BPGi and PT time (Fig 6B). In contrast, there was a significant negative correlation between circulating sTIM-3 and PT time (*p* = 0.007, r = 0.53) even in the advanced liver fibrosis stage (Fig 6A).

Serum sTIM-3 levels were significantly higher in PBC patients compared with those in healthy subjects (2395 pg/mL, IQR 2012–3422 versus. 1285 pg/mL, IQR 1098–1812, *p* <0.001). Therefore, we also investigated the correlations between serum levels of sTIM-3 and disease activity marker for PBC, such as ALP or IgM in PBC patients. Whereas we could not find any significant correlations between serum levels of sTIM-3 and ALP or IgM as well as ALT in patients with PBC (data not shown).

### Relationships between serum sTIM-3 levels and liver fibrosis and necroinflammation scores

**Fig 7:**
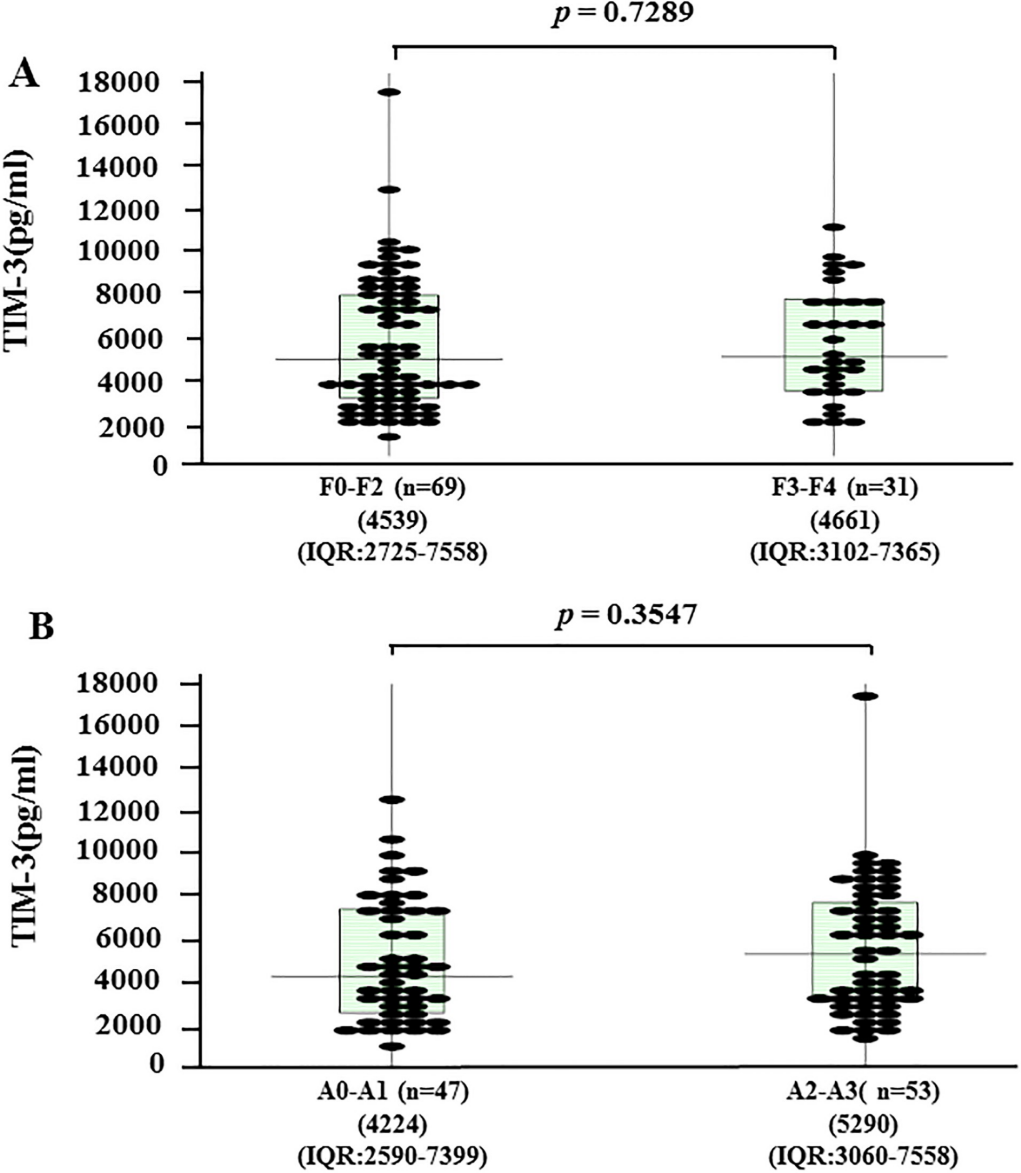
Serum levels of sTIM-3 according to liver fibrosis stage (A) and liver inflammation grade (B) in AIH patients (n = 100). The vertical lines indicate the range and the horizontal boundaries of the boxes represent the first and third quartile. Results were compared by non-parametric Mann–Whitney *U*-test.

To evaluate whether serum sTIM-3 correlated with liver histology, we grouped the AIH patients according to the liver fibrosis stage (F0–F4). The median serum concentrations of sTim-3 were 4539 pg/ml [IQR: 2725–7558] for patients at F0–F2 stage and 4661 pg/ml [IQR: 3102–7365] for those at F3-F4. There was no significant difference in serum sTIM-3 levels between these liver fibrosis stages (Fig 7A). Serum sTIM-3 values were also stratified by necroinflammatory grade (A1–A3). The median serum concentrations of sTim-3 were 4233 pg/ml [IQR: 2560–7399] for A0-A1 grades and 5290 pg/ml [IQR: 3060–7558] for A2-A3 grades. There was no significant difference in serum sTIM-3 levels according to the necroinflammatory grade (Fig 7B).

### Changes in sTIM-3 by corticosteroid therapy

**Fig 8:**
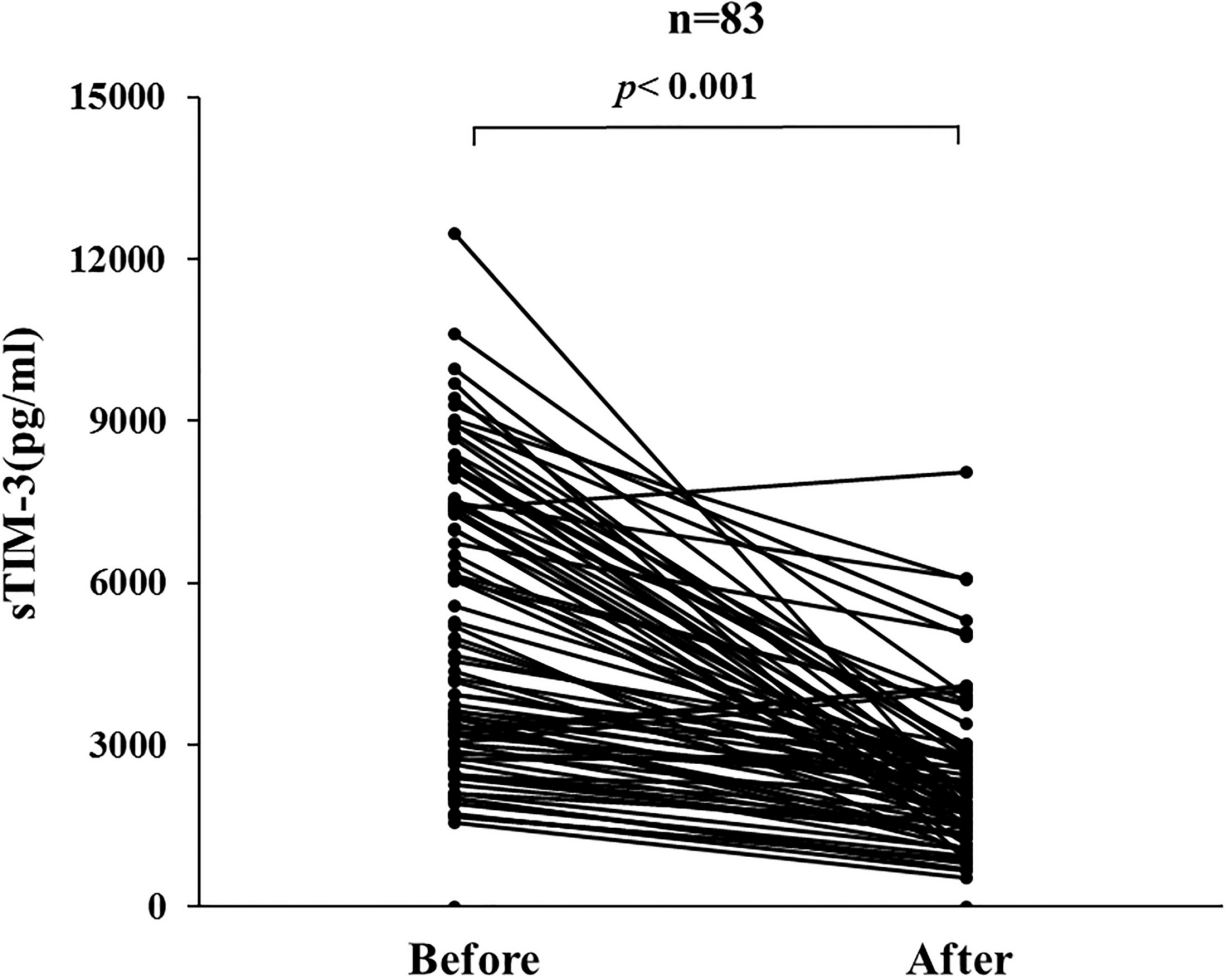
Changes in individual sTIM-3 values before and after (4weeks) steroid treatment in 83 AIH patients. Paired samples (before and after treatments) from the same AIH patients (n = 83) were compared by Wilcoxon signed-rank test.

Circulating levels of sTIM-3 were measured before and after corticosteroid therapy in paired serum samples obtained from 83 patients with AIH. Serum levels of sTIM-3 were downregulated by corticosteroid therapy, and there was a significant difference in serum levels of sTIM-3 before and after corticosteroid therapy in AIH patients (Fig 8).

## Discussion

AIH is a chronic progressive liver disease with autoimmune features that is characterized by elevated levels of aspartate aminotransferase, alanine aminotransferase and immunoglobulin G [13]. Although the etiology of AIH is obscure, environmental and genetic factors are thought to be involved in the pathogenesis of AIH [14]. A genome-wide association study on AIH revealed the strongest association with human leukocyte antigen (HLA) [15]. We found that predisposing alleles for AIH were DRB1*04:05 and DRB1*04:01 in Japanese patients with AIH [16]. HLA is responsible for the presentation of antigenic peptides to CD4^+^ T cells, which are thought to be effector lymphocytes and the main orchestrators of liver damage in AIH through the production of the pro-inflammatory cytokines [17]. Activated immune responses are kept in check by a number of protective mechanisms [18]. TIM3 is expressed on the surface of terminally differentiated T cells [19]. As a negative checkpoint receptor for T cell activation, TIM3 and its ligand galectin 9 are thought to be implicated in the pathogenesis of Th1-driven autoimmune diseases [20].

In the present study, we demonstrated that serum levels of sTIM3 were significantly higher in AIH patients compared with those in healthy subjects as well as PBC patients. Moreover, we found a positive correlation between serum sTIM3 levels and clinical parameters for liver injury, such as ALT or TB, in AIH patients. Additionally, the elevated levels of sTIM-3 were downregulated by steroid therapy, suggesting that immune-mediated hepatic inflammation could be linked with the elevated levels of sTim-3 in AIH patients. To our knowledge, this is the first study to investigate serum sTim-3 levels in patients with AIH. Our findings demonstrated that serum sTim-3 levels were significantly elevated in patients with AIH and closely associated with biochemical liver function. We previously reported that Gal-9 is elevated in AIH patients and be correlated with the degree of liver injury (AIT, total bilirubin) [21]. Similarly, the present study demonstrated that elevated levels of sTIM are correlated with the degree of liver injury in AIH. We also demonstrated that circulating sTIM-3 was negatively correlated with the hepatic spare ability (PT time, serum albumin) in AIH patients. Whereas, there was no correlation between circulating Gal-9 and these markers in AIH patients.

Co-inhibitory receptors play an important role in regulating T cell responses and immune homeostasis [22]. Interactions between TIM-3 and its ligand Gal-9 play a regulatory function in tumor immunity [23]. The dysregulation of TIM-3 expression on immune cells has shown to be associated with autoimmune diseases [24]. Our findings suggest that soluble TIM-3, by interfering with the immune regulatory activity of TIM-3 expressed on effector T cells, may play a role in the pathogenesis of AIH. However, the specific mechanism underlying TIM-3 expression in hepatic immune cells still requires further investigation.

Previous studies reported that CD4^+^ lymphocytes from AIH patients are less susceptible to Treg suppression due to reduced expression of co-inhibitory receptor TIM-3 [6]. Conversely, elevated levels of TIM-3 on CD4^+^CD25^+^ regulatory T cells can inhibit the immune-suppressive function of Treg cells, leading to IFN-γ production in the liver and exacerbating immune-mediated hepatitis [25]. The role of TIM-3 seems to be necessary to further characterize the relationship between TIM-3 expression and effector T cell function. Although serum levels of sTIM3 has not evaluated in AIH patient, elevated levels of sTIM3 were demonstrated in patients with autoimmune diseases and be correlated with disease activity [26, 27]. Similar results were obtained in this study for sTIM-3, showing its close association with disease activity of AIH. The mechanism by which TIM-3 is released from cells is not known. Because sTIM-3 is shed from immune cells expressing this molecule, the levels of serum sTIM-3 may reflex the quantity of membrane-bound TIM-3 expression on immune cells. In addition to the membrane-bound form, Tim-3 has a soluble form that is produced by cleavage from the cell surface by ADAM10 and ADAM17 [7, 28]. Although the role of membrane-bound TIM-3 against autoimmunity has been extensively documented [29], the mechanisms by which sTim-3 confers T cell exhaustion are largely unknown. In theory, sTIM-3 could act as a decoy to block the TIM-3 pathway and enhanced autoimmune responses, similar to the results of functional studies using recombinant sTim-3 performed by Jones et al [30]. However, the presence of the soluble form of receptors does not always result in blockage of the receptor function. Further studies are needed to clarify the role of sTIM-3 in immune regulation and how sTIM-3 affects this TIM-3 pathway in patients with AIH.

Another interesting finding in our study is the strongest correlation between circulating sTIM-3 and M2BPGi in patients with AIH. In consistent with the tight association to sTIM-3/M2BPGi, circulating M2PBGi was negatively correlated with the degree of liver functional reserve. Hepatic stellate cells are major producers of M2PBGi, and M2PBGi reflects the activation of hepatic stellate cells during hepatic inflammation and fibrogenesis [31]. In a clinical setting for liver injury with hepatic failure, M2PBGi production from hepatic stellate cells may be impaired. In addition to being a fibrosis or inflammatory marker, M2PBGi should be explored as a potential predictor for immune-mediated liver failure.

The dysregulation of Tim-3 expression on immune cells has shown to be associated with chronic viral infections [32]. Some reports have shown that sustained expression of TIM-3 induces deletion of viral antigen-specific cytotoxic T cells in chronic HCV infection, resulting in T cell non-responsiveness [33]. Whereas there was no significant difference in circulating sTIM-3 levels between patients with chronic HCV hepatitis and healthy controls in our study.

This study had several limitations. First, this study was relatively retrospective in nature, and the therapeutic intervention mainly depends on the participating doctors. Second, the number of study participants was relatively small. Third, given the cross-sectional design of the study, the causal relationship between sTIM-3 levels and the treatment response or clinical outcome of AIH patients could not be determined. Furthermore, liver biopsy has a limitation of sampling error for assessing the degree of fibrosis and necroinflammation.

## Conclusions

Our results showed that circulating sTIM-3 is increased and correlated with the severity of AIH. We speculate that circulating sTIM-3 could be linked immune-mediated liver damage by probably blocking the interaction between TIM-3 and its ligand, thereby abrogating the regulatory activity of TIM-3 in AIH. Functional studies are needed to better understand the biology of TIM-3 in the autoimmune reactions in liver and to determine whether TIM-3 could be a novel therapeutic target in autoimmune liver diseases.

## Supporting information

S1 File (DOCX)
